# Serum Metabolomics Coupling With Clinical Laboratory Indicators Reveal Taxonomic Features of Leukemia

**DOI:** 10.3389/fphar.2022.794042

**Published:** 2022-05-26

**Authors:** Hao- Xiong, Hui-Tao Zhang, Hong-Wen Xiao, Chun-Lan Huang, Mei-Zhou Huang

**Affiliations:** ^1^ Stem Cell Laboratory, Academician (Expert) Workstation of Sichuan Province, The Affiliated Hospital of Southwest Medical University, Luzhou, China; ^2^ Department of General Practice, The Affiliated Hospital of Southwest Medical University, Luzhou, China; ^3^ Department of Hematology, The Affiliated Hospital of Southwest Medical University, Luzhou, China

**Keywords:** leukemia, metabolomics, choline, amino acid metabolism, lipid metabolism

## Abstract

Metabolic abnormality has been considered to be the seventh characteristic in cancer cells. The potential prospect of using serum biomarkers metabolites to differentiate ALL from AML remains unclear. The purpose of our study is to probe whether the differences in metabolomics are related to clinical laboratory-related indicators. We used LC-MS-based metabolomics analysis to study 50 peripheral blood samples of leukemia patients from a single center. Then Chi-square test and T test were used to analyze the clinical characteristics, laboratory indicators and cytokines of 50 patients with leukemia. Correlation analysis was used to explore the relationship between them and the differential metabolites of different types of leukemia. Our study shows that it is feasible to better identify serum metabolic differences in different types and states of leukemia by metabolomic analysis on existing clinical diagnostic techniques. The metabolism of choline and betaine may also be significantly related to the patient’s blood lipid profile. The main enrichment pathways for distinguishing differential metabolites in different types of leukemia are amino acid metabolism and fatty acid metabolism. All these findings suggested that differential metabolites and lipid profiles might identify different types of leukemia based on existing clinical diagnostic techniques, and their rich metabolic pathways help us to better understand the physiological characteristics of leukemia.

## 1 Introduction

Acute leukemia (AL), which results from a series of mutational events that take place during the complex process of hematopoiesis, is a life-threatening hematological malignancy (Rose-Inman and Kuehl, 2017). According to the French-American-British system, AL can be classified into acute myeloid leukemia (AML) and acute lymphoblastic leukemia (ALL). Bone marrow biopsy remains the “gold standard” for the diagnosis of AML. However, the morphological, immunological and cytogenetic analysis of bone marrow biopsies is obviously invasive for patients, and the waiting time for results is long, which tends to delay the treatment of patients (Wang et al., 2019). Therefore, the discovery of early detectable non-invasive biomarkers to distinguish AML from ALL could lead to timely and accurate treatment of AL patients.

Uncontrolled growth and proliferation is a characteristic of cancer cells and requires large amounts of energy metabolites for their survival (McCartney et al., 2018). Therefore, metabolic abnormality has been considered to be the seventh characteristic in cancer cells (Hanahan and Weinberg, 2011). Metabolomics studies have shown that metabolic reprogramming was present in a variety of solid tumors including kidney cancer (Ganti and Weiss, 2011), liver cancer (Yin et al., 2009) and prostate cancer (Trock, 2011), which makes the method to be a powerful and valuable biomarker identification method (Du et al., 2018). Previous studies have revealed the metabolic differences between AL patients and normal people (Bai et al., 2014; Zhou et al., 2020). Compared with normal people, abnormal changes in multiple metabolic pathways existed in AL patients (Wang JH et al., 2013; Musharraf et al., 2017). However, few studies have mentioned the metabolomic differences between ALL and AML, and the potential value of using serum metabolites to differentiate ALL from AML remains unclear.

In this work, we used liquid chromatography-mass spectrometry (LC-MS) to analyze serum metabolites from 50 patients with a clear diagnosis of AL. Twenty seven out of metabolites were found differentiative among ALL and AML patients using *p*-value <0.05. We expected to distinguish AML from ALL by metabolomics, and we hypothesized that metabolites differentially expressing between the two groups were related biomarkers. Then we used different methods to detect the clinical laboratory indicators of the samples, and further explored the relationship between it and metabolomics. The research aimed to investigate the differences in serum metabolites in subjects with different types of leukemia and to explore the related metabolic pathways between AML and ALL, so that we can better understand the metabolic characteristics and potential mechanisms of acute leukemia. Moreover, the finding of potential biomarkers is helpful to diagnose and monitor acute leukemia, as well as ultimately develop new drugs for therapeutic interventions.

## 2 Materials and Methods

### 2.1 Patients and Specimen Collection

Serum specimens of patients with acute leukemia admitted to our department from May to November 2020 were collected. This study was approved by the Ethics Committee of the Affiliated Hospital of Southwest Medical University (approval number: 2020250). The diagnosis was according to the 2008 WHO criteria (Vardiman et al., 2009). We selected newly diagnosed patients, or revisited patients whose efficacy was assessed as unremission or partial remission, their MRD (Minimal residual disease) are all non-negative. The exclusion criteria for selected AML patients are as follows: 1) Patients with metabolic diseases such as hepatitis, diabetes, renal failure and hyperthyroidism, 2) Patients with acute or chronic respiratory failure, 3) Patients who still need vasoactive drugs to maintain after active fluid resuscitation after shock, 4) Patients requiring mechanical ventilation for endotracheal intubation, 5) Acute leukemia patients secondary to chemotherapy, radiotherapy, myelodysplastic syndrome and myeloproliferative disease, 6) With other malignant tumors, 7) Patients have received hematopoietic stem cell transplantation, 8) Patients who refused to participate in the study. According to the above criteria, we strictly screened 50 patients with acute leukemia, including 14 cases of acute lymphoblastic leukemia and others are acute myeloid leukemia. According to the Declaration of Helsinki, all participants have obtained written informed consent prior to clinical recording and sample collection. Serums from all patients were collected before starting treatment. Blood routine, biochemical tests and cytokine detection were performed on all patients while the supernatant was collected. The cytogenetics and gene expression status of these 50 patients at the time of initial diagnosis were obtained by consulting the hospital’s electronic medical record system. For prognostic grouping based on cytogenetics, ALL patients use the grouping criteria for prognostic factors of adult acute lymphoblastic leukemia (Rowe, 2010), and AML patients use the Cancer and Leukemia Group B (CALGB) grouping standard (Heilmeier et al., 2007). When comparing the differences between the two groups of AML patients, we adopted the 2017 European Leukemia Network (ELN) risk stratification recommendations (Dohner et al., 2017).

After fasting overnight, peripheral blood samples were collected from all participants and yellow-headed tubes containing inert separation glue and coagulants were used. The blood samples were centrifuged at 3000 rpm and 4°C for 10 min. Serum samples were obtained and stored at −80°C until further use.

### 2.2 Metabolite Samples Preparation

For LC–MS metabolomic analysis, 100 μL serum sample were mixed with 200 μL ice-cold acetonitrile, and then vortexing the mixture for 30 s, incubating the mixture at –20°C for 10 min and then 12000× g centrifuging the mixture for 10 min at 4°C. 20 μL serum from each sample were mixed to prepare a quality control (QC) sample. Then, the mixture was divided into aliquots with the same volume as other samples and prepared together. The supernatant after centrifugation was filtered through a 0.2 µm nylon mesh into sample vials to LC/MS analysis.

### 2.3 UPLC/QTOF-MS Analysis of Serum Metabolites

The metabolomics analysis was performed with an Agilent 1290 Infinity LC system coupled to an Agilent 6530 Accurate-mass Q-TOF mass spectrometer (Agilent Technologies, Palo Alto, CA, United States). Chromatographic separation of serum samples was performed on an Agilent ZORBAX SB-C18 threaded column (2.1 × 150 mm, 1.8 µm, Agilent Technologies, Palo Alto, CA, United States) maintained at 35°C. The mobile phase consisted of solvent A (0.1% formic acid in water, v/v) and B (0.1% formic acid in acetonitrile, v/v). The optimized gradient program was established. The post time was set to 3 min for equilibration. Mass spectrometry was performed in both positive (ESI+) and negative (ESI-) electrospray ionization modes. The fragment voltage was set to 135 V and the skimmer voltage was set to 65 V. The capillary voltages were set to 4.0 KV in positive mode and 3.5 KV in negative mode. The drying gas (nitrogen) was used at a flow rate of 10 L/min at 350°C and the nebulizer pressure was set to 45 psig. Data were collected in centroid mode from 50 to 1000 m/z using an extended dynamic model.

Serum samples were analyzed in random order during the analysis. In addition, QC samples were detected once every 6 subject samples for conditioning of the analytical system, signal correction, and quality assurance.

### 2.4 Flow Cytometry

Heparin anticoagulation tubes were used tocollect approximately 2 ml of the patient’s bone marrow fluid at the time of the bone marrow aspiration. The specimen was placed in a special tube for flow cytometry and hemolysin was added, lysis of erythrocytes in the sample for 10min. The lysed specimen was centrifuged at 300 g for 5 min, the supernatant was discarded and 1 ml of PBS solution was added for washing and repeated. After the erythrocyte lysis is complete the appropriate antibody is added to the flow tube, incubate for 15 min at low light and then assay using a Beckman flow cytometer. We used winMDI software for the analysis of the results, 2000 cells per flow tube were analysed and phenotypic analysis was performed with a CD45/ssc two-parameter two-site scatter plot gating.

### 2.5 Bone Marrow Blast Cell Count Analysis

The patient was placed in the prone position and fully disinfected. The posterior superior iliac spine was selected as the puncture site. After local anesthesia with 2% lidocaine, 0.2 ml of bone marrow fluid was extracted to make a uniform thickness of bone marrow smear, and the prepared bone marrow smear was stained with Swiss Giemusa. The proliferation of bone marrow nucleated cells was observed under low power microscope. Then 200 nucleated cells were counted and analyzed under oil microscope, and the proportion of primitive cells was calculated and recorded.

### 2.6 Blood Routine Analysis

While collecting test samples, we also used purple blood vessels without anticoagulants to extract the peripheral blood of 50 patients with AML, and used automatic blood routine analyzer (Japan, XT-2000i) to perform routine blood analysis on the peripheral blood of the patients. The neutrophil count (NEU), hemoglobin content (Hb) and platelet (PLT) values were recorded after analysis.

### 2.7 Serum Biochemistry Analysis

The serum concentrations of Lactate dehydrogenase (LDH), alkaline phosphatase (ALP), Calcium ions (Ca+), Creatinine, albumin (ALB), globulin (GLB), Triglycerides, Cholesterol, Low density lipoprotein (LDL), High density lipoprotein (HDL), Apolipoprotein A1,Apolipoprotein B, were detected by an automatic biochemical analyzer (The United States, Beckman) according to the manufacturer’s instructions for the corresponding commercial kits.

### 2.8 Cytokine Detection

Standard materials with different concentration gradient were prepared according to the instructions. Mixtures of captured microspheres were prepared according to the requirements. Add 25 μL of the mixture of captured microspheres to the test tube, add 25 μL of sample (fresh serum) to the sample tube, and add 25 μL of the gradient diluted standard to the Standard Quality Control (QC). After all the experimental tubes were fully mixed, they were incubated at room temperature for 1 h in a dark environment. Add 25 μL fluorescence detection reagents (C) to each tube, stir gently and incubate at room temperature for 2 h. Add 1 ml PBS solution to the test tube, centrifuge at 200 g for 5 min, then carefully suck the supernatant, add 100 μL PBS solutions, and stand for detection. Fluorescence detection was performed on a well-calibrated flow cytometer (The United States, Beckman) in the order of standard quality control, negative tube, and sample tube. Each test tube is required to be tested immediately after vortex blending for 3–5 s. According to the instructions, calculate the corresponding value according to the concentration gradient corresponding to the dilution factor of the standard.

### 2.9 Statistical Analysis

#### 2.9.1 Multivariate Statistical Analysis of Plasma Metabolite Data

The raw MS data was converted by abfConverter (Nonlinear Dynamics, Newcastle, UK). The converted MS data was imported into MSDIAL software to filter the noise, calibrate the baseline, align the peaks, and identify and quantify the peaks. Retention time deviations of less than 0.15 min were allowed to align the peaks. Ion peaks with missing values >50% in both groups were deleted from the alignment data. Then, the normalized data with auto-scaling were imported into SIMCA-P (version 13.0, Umetrics AB, Umea, Sweden), to perform multivariate and single-dimensional statistical analysis, including unsupervised principal component analysis (PCA), supervised Orthogonal partial least squares discriminant analysis (OPLS-DA), t-test, and fold change analysis. The potential biomarkers were selected in accordance with variable importance in projection (VIP) score >1 from the OPLS-DA model. The potential biomarkers were further optimized by Student’s t-test for their abundance in different groups. Adjusted *p* value <0.05 was considered to be statistically significant. The biomarkers were further screened in accordance with VIP score >1, adjusted *p* value <0.05, and fold change >1.5 or <0.67.

#### 2.9.2 Analysis of Clinical Laboratory Indicators

SPSS 20 was used for statistical analysis, we used chi-square test and independent sample t-test to analyze the clinical characteristics, cytogenetic prognostic groupings at first diagnosis, common laboratory indicators and cytokine of 50 leukemia patients in different groups, *p* value <0.05 considered the difference to be statistically significant. Then use correlation analysis to explore the relationship between clinical laboratory indicators and different metabolites that have differences between groups, *p* value <0.05 is statistically significant.

### 2.10 Biomarker Identification and Metabolic Pathway Analysis

Metabolites were identified through a mass/mass-based search followed by manual verification. Accurate mass values of the molecular ions of TOF-MS data were matched against METLIN and Human Metabolome Database (HMDB). Then, an MS/MS analysis was conducted to confirm the structure of potential biomarkers by matching the masses of the fragments. The parent ion mass tolerance is ±10 ppm and mass/charge (m/z) of products tolerance is ±10 ppm. Cluster analysis of the potential biomarkers was performed by R (version 3.6.1) and the metabolic pathways were identified using the KEGG database.

## 3 Results

### 3.1 Clinical Features of 50 Samples of Acute Leukemia Patients

The 50 cases of acute leukemia include 14 cases of acute lymphoblastic leukemia and 36 cases of acute myeloid leukemia. The average age of 50 leukemia patients is 46 years and a median age of 48 years, Including 27 cases of males with an average age of 47.8 ± 21.1 years, and 23 cases of females with an average age of 44.0 ± 19.5 years. The basic blood routine and biochemical indexes of the patient are shown in Table 1; Table,2.

**TABLE 1:** Comparison of demographics, prognosis grouping, cytokines and clinical details between AML and ALL patients (Means ± SD).

Index	ALL (*n* = 14)	AML (*n* = 36)	χ^2^/t	*p* value
Gender, (male/female)	7/7	20/16	0.125	0.723
Age (years)	41.79 ± 20.70	47.69 ± 20.11	−0.925	0.359
BMI	19.15 ± 2.96	20.41 ± 2.65	−1.460	0.151
Prognosis grouping (favorable/intermediate/adverse)	3/9/2	7/16/13	2.414	0.299
Bone marrow blasts (%)	46.09 ± 35.20	45.17 ± 36.57	0.071	0.944
IL-4	1.30 ± 1.05	1.85 ± 1.39	−1.358	0.181
IL-10	16.11 ± 19.02	20.27 ± 18.75	−0.309	0.759
NEU	1.31 ± 1.78	2.70 ± 7.79	−0.610	0.545
HGB	74.92 ± 16.12	76.38 ± 18.45	−0.244	0.808
PLT	48.75 ± 40.12	34.41 ± 38.66	1.094	0.280
LDH	368.49 ± 395.16	688.39 ± 138.78	−0.772	0.445
ALP	95.71 ± 59.34	78.35 ± 41.98	1.165	0.250
Ca^2+^	2.19 ± 0.25	2.10 ± 0.19	1.466	0.149
Creatinine	67.87 ± 36.16	61.80 ± 23.30	0.704	0.485
Albumin	38.08 ± 7.23	34.68 ± 5.56	1.680	0.100
Globulin	22.50 ± 3.86	24.58 ± 4.90	−1.426	0.160
Triglycerides	5.34 ± 8.51	1.32 ± 0.80	2.617	0.013^*^
Cholesterol	3.82 ± 1.36	3.08 ± 2.20	1.619	0.114
LDL	2.17 ± 1.00	1.91 ± 0.93	0.754	0.455
HDL	1.09 ± 0.43	0.78 ± 0.34	2.304	0.027^*^
Apolipoprotein A1	1.01 ± 0.38	0.88 ± 0.34	1.020	0.314
Apolipoprotein B	0.82 ± 0.35	0.67 ± 0.28	1.371	0.179

*P < 0.05.

BMI, body mass index; IL-4, interleukin 4; IL-10, interleukin 10; NEU, neutrophils; HGB, hemoglobin; PLT, platelets; LDH, lactate dehydrogenase; ALP, alkaline phosphatase; LDL, low density lipoprotein; HDL, high density lipoprotein.

**TABLE 2:** Comparison of demographics, prognosis grouping, cytokines and clinical details between the two groups of AML (Means ± SD).

Index	AML group C (*n* = 16)	AML group B (*n* = 20)	χ^2^/t	*p* value
Gender, (male/female)	11/5	9/11	2.031	0.154
Age (years)	47.19 ± 17.14	48.10 ± 22.65	−0.133	0.895
FAB type, (M1/M2/M3/M4/M5)	3/3/4/2/4	2/6/4/3/5	1.080	0.897
BMI	20.34 ± 2.62	20.45 ± 2.75	−0.127	0.899
Bone marrow blasts (%)	53.58 ± 33.03	37.36 ± 39.13	1.159	0.257
Prognosis grouping (favorable/intermediate/adverse)	3/8/5	3/13/4	0.868	0.648
IL-4	2.19 ± 1.40	1.59 ± 1.35	1.300	0.202
IL-10	29.77 ± 71.19	12.66 ± 15.47	1.048	0.302
NEU	4.06 ± 10.96	1.63 ± 3.87	0.901	0.374
HGB	78.20 ± 22.60	74.95 ± 14.89	0.505	0.617
PLT	26.33 ± 26.51	40.79 ± 45.78	−1.086	0.286
LDH	370.00 ± 258.18	982.29 ± 1824.4	−1.150	0.262
ALP	65.51 ± 25.37	88.62 ± 49.88	−1.684	0.101
Ca^2+^	2.09 ± 0.14	2.10 ± 0.22	−0.053	0.958
Creatinine	61.74 ± 22.58	61.85 ± 24.45	−0.014	0.989
Albumin	34.81 ± 5.88	34.58 ± 5.45	0.117	0.908
Globulin	24.56 ± 3.81	24.60 ± 5.72	−0.023	0.982
Triglycerides	1.03 ± 0.62	1.54 ± 0.86	-1.791	0.084
Cholesterol	3.17 ± 1.46	3.02 ± 1.00	0.336	0.739
LDL	2.05 ± 1.11	1.80 ± 0.79	0.700	0.490
HDL	0.79 ± 0.39	0.77 ± 0.32	0.188	0.852
Apolipoprotein A1	0.87 ± 0.40	0.90 ± 0.29	−0.233	0.818
Apolipoprotein B	0.71 ± 0.35	0.64 ± 0.21	0.678	0.504

BMI, body mass index; IL-4, interleukin 4; IL-10, interleukin 10; NEU, neutrophils; HGB, hemoglobin; PLT, platelets; LDH, lactate dehydrogenase; ALP, alkaline phosphatase; LDL, low density lipoprotein; HDL, high density lipoprotein.

### 3.2 Metabonomics Differences Between Subjects With Acute Leukemia

**FIGURE 1:**
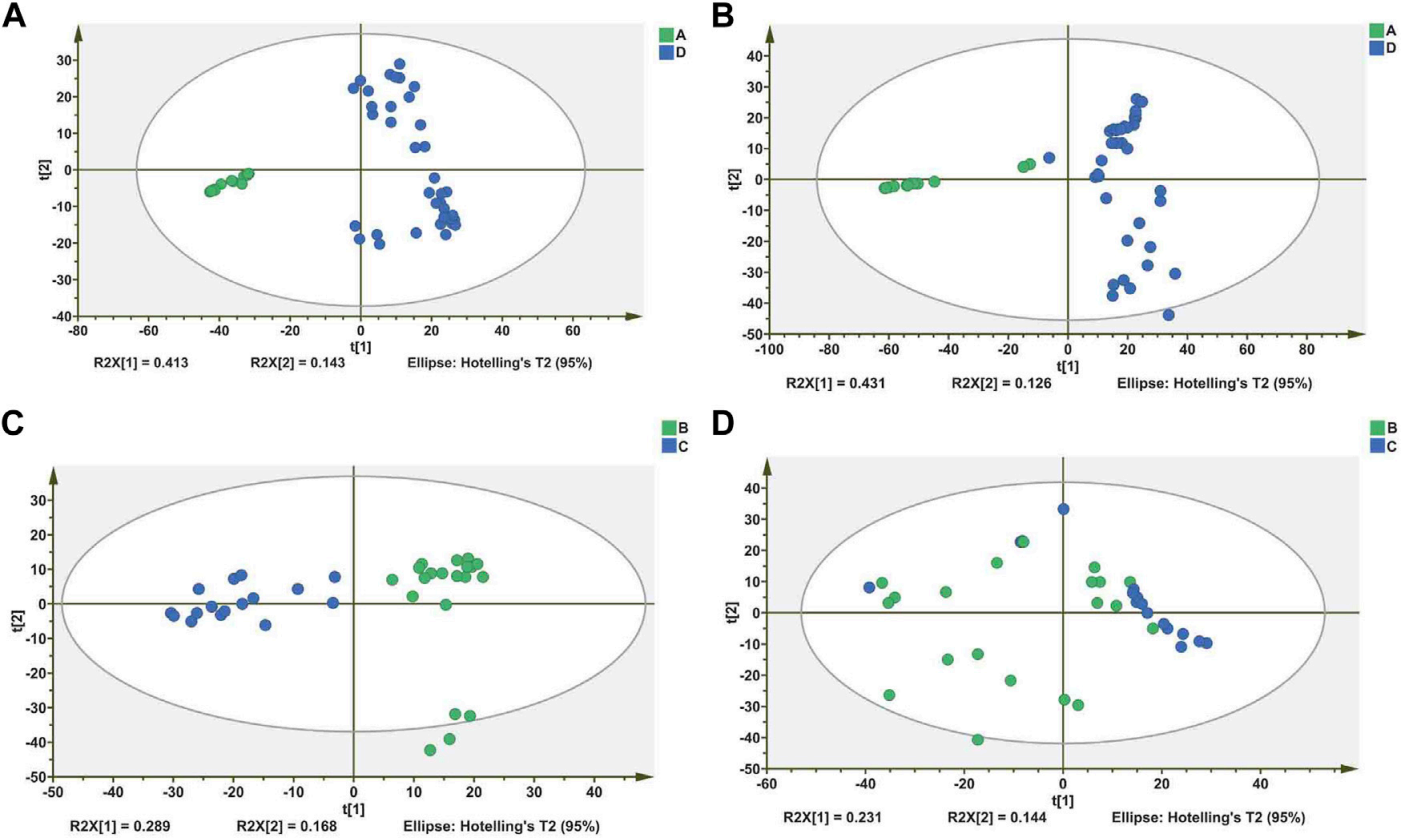
Differentiation of metabolic profiles of serum of patients with different types of leukemia. **(A,C)**: Principal component analysis (PCA) score plots based on serum metabolic profiles of Acute lymphoblastic leukemia (ALL) and Acute myeloid leukemia (AML) groups in positive and negative modes. **(B,D)** PCA score plots based on serum metabolic profiles of within the AML group in positive and negative modes.

To understand the metabolic differences between different acute leukemia, we performed a metabolomic analysis of sera from patients with acute leukemia. PCA, which is an unsupervised pattern recognition analytical method based on the LC-MS data, was then used to visualize the trends among the groups. In the PCA scores, each point represents an individual sample, and significant differences between patients with acute leukemia can be observed as shown in Figures 1A,B. The model R2X parameters, which represent the model’s ability to interpret variables, were determined to be at 0.413 and 0.431 in the positive and negative ion mode, respectively, which indicates that 41.3 and 43.1% of the variables are used in building the analysis model in the positive and negative mode, respectively. In the positive and negative ion mode, the acute leukemia plasma PCA score could be significantly separated into two groups (group A and group D), suggesting the existence of serum metabolic disorder between the two groups of acute leukemia patients. Combined with the clinical data of the patients, we found that patients in group A were acute lymphoblastic leukemia and patients in group D were acute myeloid leukemia. In other words, there are significant metabolic differences between patients with acute lymphoblastic leukemia and patients with acute myeloid leukemia. Patients with acute myeloid leukemia also showed significant clustering (group B and group C), so we also analyzed the serum metabolites of 36 patients with AML, and the PCA results were analyzed as shown in Figures 1C,D.

**FIGURE 2:**
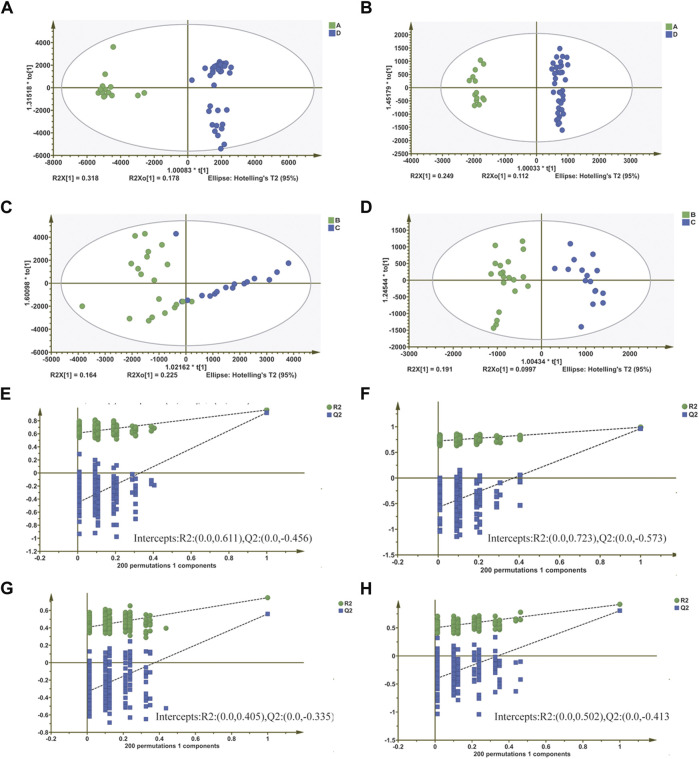
Screening of differential metabolites based on orthogonal partial least squares discriminant analysis (OPLS-DA) model. **(A,B)** OPLS-DA score plots based on serum metabolic profiles of ALL and AML groups in positive and negative modes. **(C,D)** OPLS-DA score plots based on serum metabolic profiles of within the AML group in positive and negative modes. **(E–H)** Permutation test of OPLS-DA model for different comparison groups.

To improve the classification, we introduced OPLS-DA. The OPLS-DA score showed significant separation of ALL and AML (Figures 2A,B), indicating significant metabolic differences between them, so as the AML groups (Figures 2G,H). This finding is consistent with the PCA results. Score plots, which supervised the OPLS-DA analysis, also used in our analysis. The Score plots showed obvious separation between AML and ALL in both positive (Figure 2C) and negative (Figure 2D) ion modes, suggesting biochemical perturbation between AML and ALL due to the disease. The Score plots analyzed for AML also drew such a conclusion. Further, in order to ensure the reliability of the results, permutation test (*n* = 200) was used to verify the OPLS-DA model of metabolomics analysis. In the analysis of ALL and AML, the intercepts of R2 and Q2 parameters reached 0.611 and 0.456, respectively, in positive ion mode, and 0.723 and 0.573, respectively, in negative ion mode (Figures 2E,F). In the analysis of AML, the intercepts of R2 and Q2 parameters reached 0.405 and 0.335 respectively in positive ion mode, and 0.505 and 0.413 respectively in negative ion mode (Figures 2K,L). All of those results further indicate that no overfitting has occurred in our model.

### 3.3 Identification of Differential Metabolites

**FIGURE 3:**
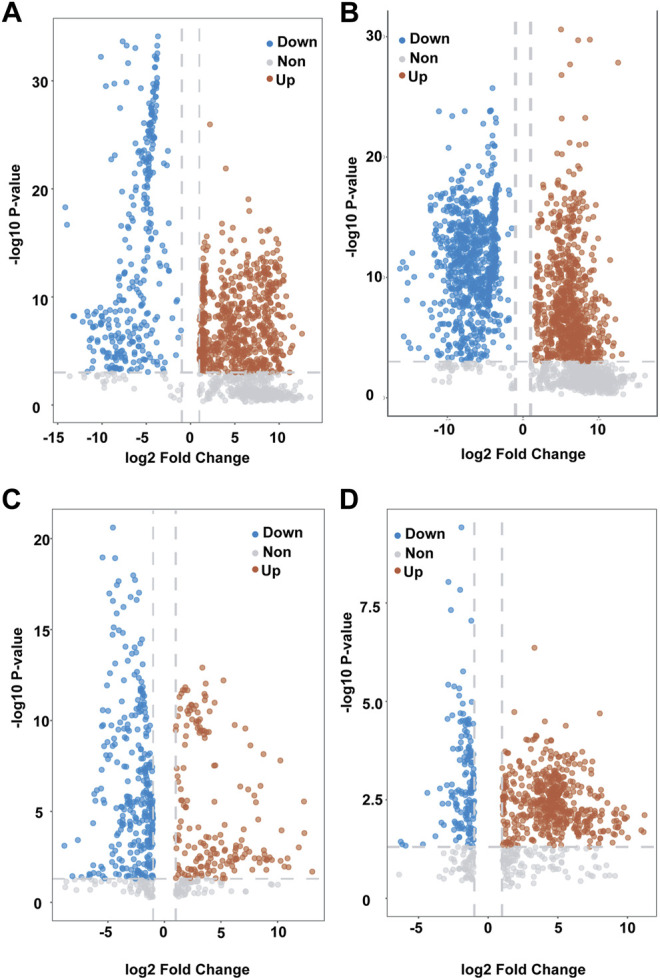
Candidate biomarkers in serum of patients with different types of leukemia. **(A,B)**: Differential metabolite volcanic map between group A and group D in negative/positive mode.**(C,D)**: Differential metabolite volcanic map between B and group C in negative/positive mode. Group A Acute lymphoblastic leukemia group D.(include group B and group C): Acute myeloid leukemia.

**FIGURE 4:**
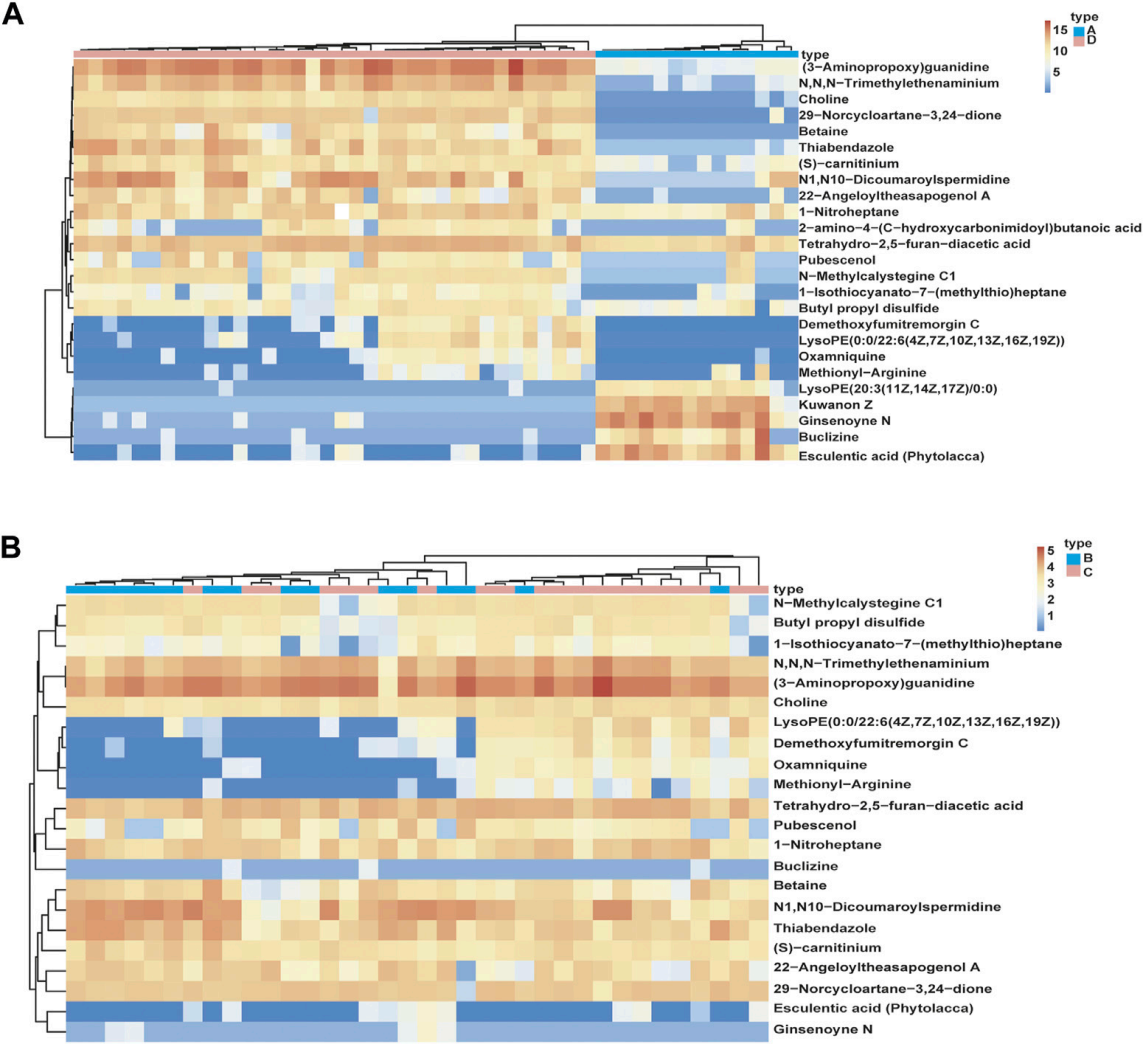
Relationship among different types of leukemia samples, and expression patterns of potential biomarkers in different samples. **(A)**: Relationship between ALL and AML samples, and expression patterns of potential biomarkers in different samples. **(B)**: Relationship within AML samples. Group A: Acute lymphoblastic leukemia (ALL), group D (include group B and group C): Acute myeloid leukemia (AML).

In order to identify different metabolites in the serum of patients with AL, metabolic analysis software and volcano maps were used. We set at FDR<0.05 as the significance threshold to control the rate of false discovery. The VIP value, which was obtained from the OPLS-DA model, was used to identify metabolites using Student’s t-test to ensure that the metabolites selected were statistically significant. The compounds for which the VIP value was >1 and *p* < 0.05 were screened. Twenty-seven metabolites (18 positive patterns and 9 negative patterns) had the ability to distinguish between the AML and ALL groups (groups A and D) (Figures 3A,B, Figure,4A), and 24 metabolites (16 positive patterns and 8 negative patterns) divided AML into two groups (groups B and C) (Figures 3C,D, Figure 4B). We used the MS/MS fragment patterns in HMDB to search for these potential biomarkers, which include choline, betaine, pubescenol, Butyl propyl disulfide and other metabolites as shown in Table 3.

**TABLE 3:** Differences in serum metabolites between groups of ALL and AML and between groups of acute myeloid leukemia.

**Metabolites**	***p* value (A/D)**	**FC (A/D)**	***p* value (B/C)**	**FC (B/C)**
Choline	<0.01	622.77	0.41	1.08
Betaine	<0.01	1159.03	0.03	0.48
Butyl propyl disulfide	<0.01	7.71	0.03	0.64
(3-Aminopropoxy)guanidine	<0.01	4.46	0.51	0.86
1-Isothiocyanato-7-(methylthio) heptane	<0.01	19.65	0.05	0.46
1-Nitroheptane	<0.01	5.48	<0.01	0.44
(S)-carnitinium	<0.01	0.122	0.27	0.13
Tetrahydro-2,5-furan-diacetic acid	<0.01	2.97	<0.01	0.59
N-Methylcalystegine C1	<0.01	367.20	<0.01	1.48
Pubescenol	<0.01	239.92	0.03	2.33
N,N,N-Trimethylethenaminium	<0.01	355.02	0.06	1.52
(3-Aminopropoxy)guanidine	<0.01	282.53	0.02	1.59
Thiabendazole	<0.01	428.09	<0.01	0.26
(S)-carnitinium	<0.01	18.07	0.02	0.62
Oxamniquine	0.02	207.85	<0.01	34.26
Methionyl-Arginine	0.55	0.63	0.02	43.00
Demethoxyfumitremorgin C	0.03	687.37	<0.01	69.46
29-Norcycloartane-3,24-dione	<0.01	240.23	0.41	1.07
Buclizine	0.02	0.41	0.91	1.06
N1,N10-Dicoumaroylspermidine	<0.01	9.95	0.04	0.61
Ginsenoyne N	<0.01	0.32	0.89	1.16
Esculentic acid (Phytolacca)	<0.01	0.09	0.92	1.06
LysoPE (20:3 (11Z,14Z,17Z)/0:0)	<0.01	0.04	—	—
LysoPE (0:0/22:6 (4Z,7Z,10Z,13Z,16Z,19Z))	0.02	1137.59	<0.01	23.59
22-Angeloyltheasapogenol A	<0.01	103.01	<0.01	0.53
Kuwanon Z	<0.01	0.002	—	—

Group A: Acute lymphoblastic leukemia, group D (include group B and group C):Acute myeloid leukemia. FC, fold changes.

### 3.4 Detection of Leukocyte Surface Antigen Expression in Bone Marrow Samples by Flow Cytometry

Flow cytometry is a technique for the quantitative analysis and sorting of chemical components on the surface of individual cells or within cells technique. It has the advantages of speed, sensitivity and accuracy, and a wide range of parameters, which are important in the diagnosis and differential diagnosis of hematological tumors. We used flow cytometry to label the expression of CD34, CD117, CD13, CD33, CD15 surface antigens in leukocytes from bone marrow samples of these 50 patients and analysed them statistically. For comparison with sample subgroups obtained using serum metabolomics analysis.

**FIGURE 5:**
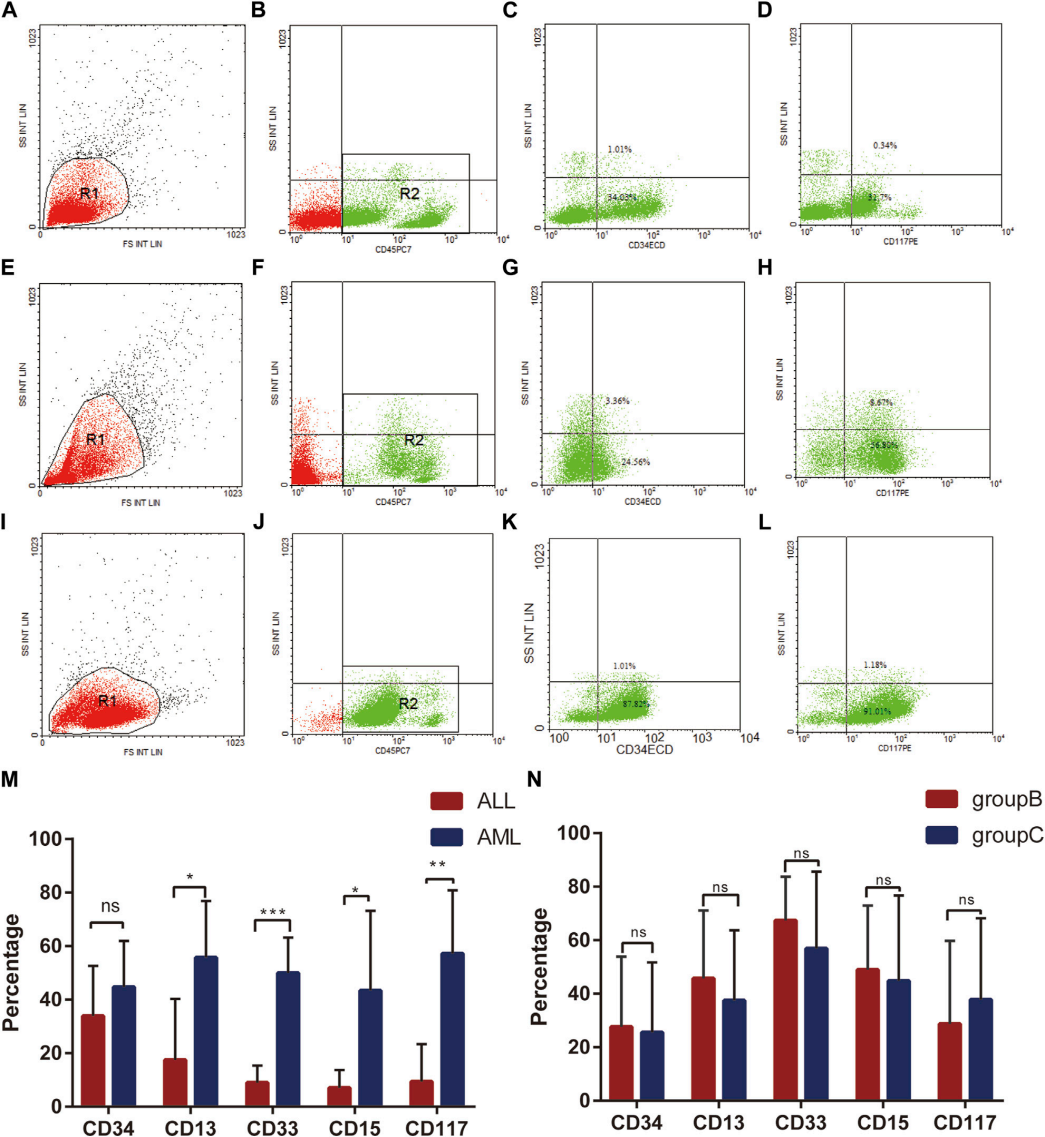
Leukocyte surface antigen expression detected by flow cytometry. **(A–D)**: Expression of leukocyte surface antigen CD34 **(C)**, CD117 **(D)** in acute lymphoblastic leukemia (group A); **(E–H)**: Expression of leukocyte surface antigen CD34 **(G)**, CD117 H in acute myeloid leukemia (group B); **(I–L)**: Expression of leukocyte surface antigen CD34 **(K)**, CD117 **(L)** in acute myeloid leukemia (group C); **(M)**: Expression of leukocyte surface antigens CD34, CD117, CD13, CD33, CD15 in AML (group D) and ALL (group A). **(N)**: Expression of leukocyte surface antigens CD34, CD117, CD13, CD33, CD15 between AML groups (groups B and C).

The results showed no significant difference in the expression rate ofantigen CD34 in patients with AML and ALL. Expression rates of CD117, a myeloid-specific marker antigen, differ significantly between AML and ALL patients (Figure 5, Supplementary Figure.S1). The expression rates of antigens CD13 and CD33 and antigen CD15 were significantly higher in AML compared to ALL, with statistically significant differences (Figure 5, Supplementary Figure.S1). This also validates the reliability of the results of the subgroup of leukaemia patients by serum metabolomics analysis. In contrast, in the two groups of AML patients separated by serum metabolomics, there were no significant differences in the expression rates of CD34, CD13, CD33, CD15, or CD117 antigens (Figure 5, Supplementary Figure.S1).

### 3.5 Comparison of Clinical Characteristics and Laboratory Indexes Among Different Groups of 50 Leukemia Patients

**FIGURE 6:**
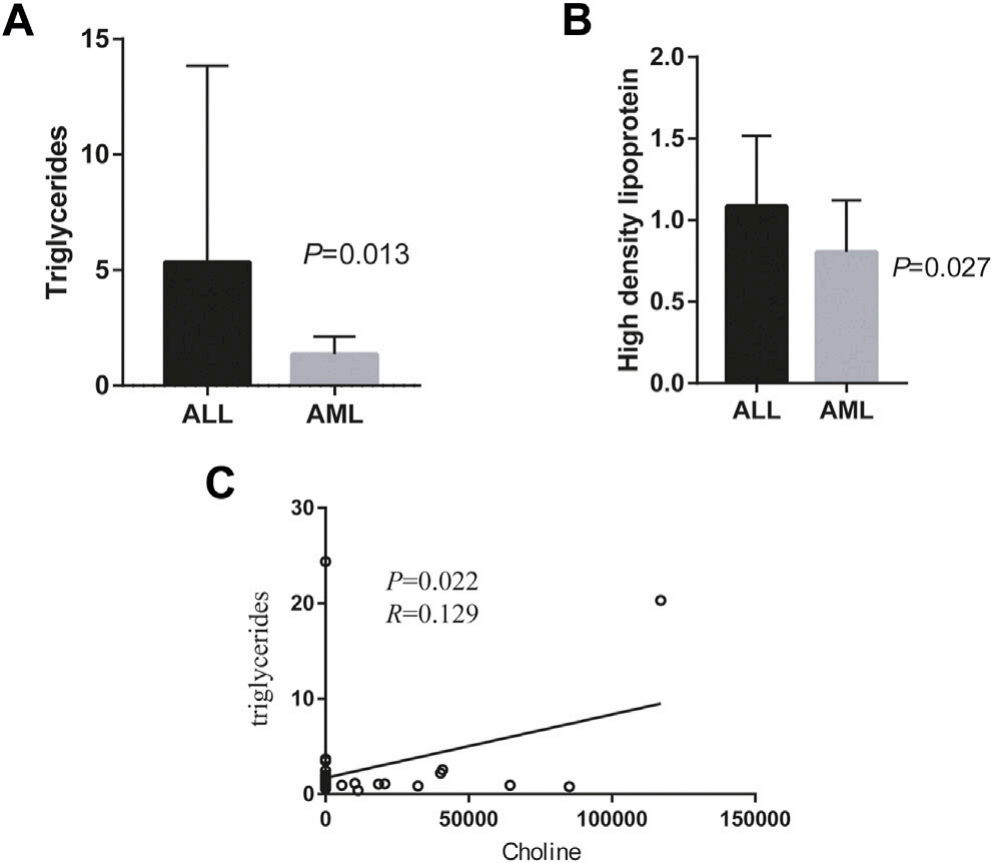
Comparison of clinical data and correlation analysis of metabolites between different leukemia groups. **(A)**: Comparison of High density lipoprotein between acute lymphoblastic leukemia and acute myeloid leukemia; **(B)**: Comparison of High density lipoprotein between acute lymphoblastic leukemia and acute myeloid leukemia; **(C)**: Comparison of High density lipoprotein and choline.

We used chi-square test and T test to compare the demographic characteristics and clinical details of these 50 patients in different groups. The differences in triglycerides and high-density lipoproteins between 14 patients with acute lymphoblastic leukemia and 36 patients with acute myeloid leukemia were statistically significant. There was no statistical difference between the two groups in laboratory indicators such as gender, age, BMI, cytogenetic prognostic groupings at first diagnosis, the percentage of bone marrow blasts, albumin, globulin, peripheral blood leukocytes, hemoglobin, platelets, etc (Table 1; Figures 6A,B). Among the 36 patients with AML, there was no significant difference in demographic characteristics and cytogenetic prognostic groupings at first diagnosis, clinical test indicators between group B and group C (Table2).

### 3.6 Comparison of Cytokines Related to Tumor Burden Among Different Groups of 50 Leukemia Patients

We also used an independent sample T test to compare the cytokines interleukin 4 (IL-4) and interleukin 10 (IL-10) between different groups of these 50 leukemia patients, which were obtained by flow cytometry as stated in the previous complaint. Unfortunately, in these 50 patients with leukemia, there was no significant statistical difference in the expression of cytokines IL-4 and IL-10 between different groups (Table1 and Table2).

### 3.7 The Relationship Between Different Metabolites and Different Clinical Laboratory Indexes and Cytokines in Different Groups of 50 Cases of Leukemia

Then we analyzed the correlation between these different metabolites and different clinical test and the cytokines, and found that there was a significant positive correlation between choline and triglycerides (*p* < 0.05), as shown in Figure 6C. The relationship between choline and betaine and cholesterol, high-density lipoprotein, low-density lipoprotein, apolipoprotein A1, apolipoprotein B and cytokines is shown in additional files Figures 2,3. We did not find a significant correlation between them (*p* > 0.05).

### 3.8 Acute Leukemia-Related Metabolic Pathways

**FIGURE 7:**
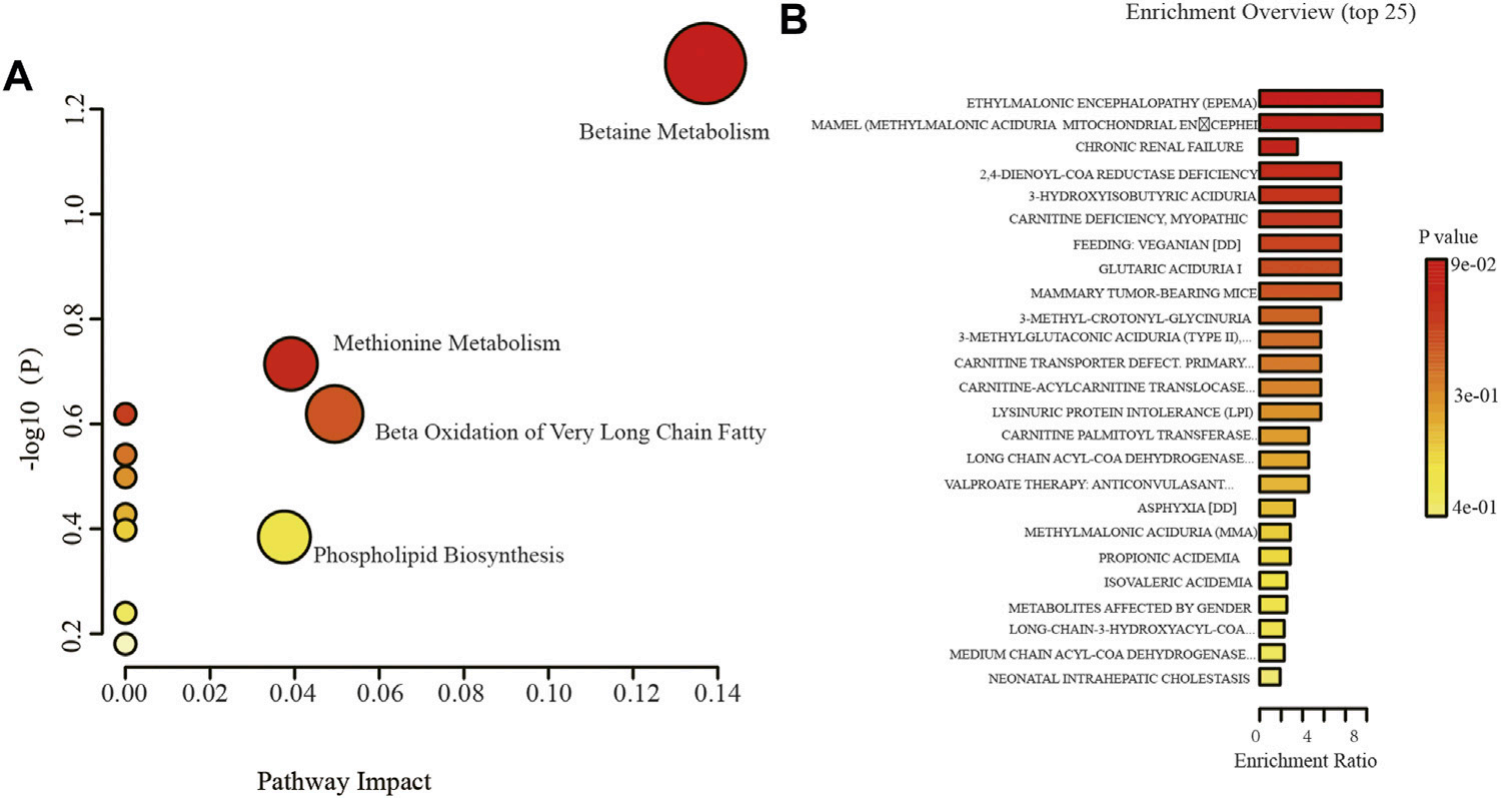
The enrichment analysis of potential biomarkers. **(A)**: KEGG pathway enrichment analysis of potential biomarkers, **(B)**: Disease-related pathway enrichment analysis of potential biomarkers.

The effect of these screened differential metabolites on metabolic pathways is of interest to us. Therefore, in order to test the influence of these differential metabolites on metabolic pathways, the KEGG pathway enrichment analysis was performed on the differential metabolites that could be identified in the KEGG database. As shown in Figure 7A, these metabolites are involved in betaine metabolism, glycine and serine metabolism, methionine metabolism, phospholipid biosynthesis, beta oxidation of very long chain fatty acids and other metabolic pathways.

Further, we compared and enriched these differential metabolites with Disease associated metabolite sets (reported in blood) by using MetaboAnalyst 5.0 software. The results showed that ethylmalonic encephalopathy, chronic renal failure, 2,4-dienoyl-coa reductase deficiency, carnitine deficiency, etc. were involved in the metabolic pathways of disease. We also analyzed the KEGG metabolic pathway that these differential metabolites participate in, and the results showed that the pathways involved in differential metabolites include fatty acid, phospholipid metabolism, etc, as shown in Figure 7B.

## 4 Discussion

In recent reports, metabolomics techniques have been used to study acute leukemia. There are few studies to explore the metabolomics differences between ALL and AML. Meanwhile, the potential value of using serum metabolites to differentiate ALL from AML still remains unclear. It was not reported whether the differences in metabolomics were related to clinical laboratory-related indicators and cytokines. In our research, we studied 50 peripheral serum samples including 14 adult ALL patients and 36 adult AML patients using a metabolomics approach. We used permutation test to verify the OPLS-DA model of metabolomics analysis. Results indicate that no overfitting occurred in our model. Therefore, the multivariate statistical model should be effective. And we also analyzed the bone marrow cells of these patients using flow cytometry. The primitive cell antigen CD34, myeloid-specific marker antigen CD117, myeloid early differentiation antigens CD13 and CD33, and myeloid late differentiation antigen CD15 were selected as surface antigen markers of leukocytes to validate the sample grouping obtained by serum metabolomics analysis (Gorczyca et al., 2011; Sandes et al., 2013). The reliability of serum metabolomics to differentiate leukemia types was confirmed. Our research shows that there are indeed differences in the metabolism between acute myeloid leukemia and acute lymphoblastic leukemia, and these differences may be related to clinical test indicators.

In Table 3, we see that the levels of choline and betaine in the serum of patients with acute myeloid leukemia are significantly higher than those in lymphocytic leukemia. In recent years, abnormal choline metabolism also has emerged as consistent hallmarks of cancer, and can be considered as a new target for cancer treatment (Glunde et al., 2011). Changes in choline kinase-a, ethanolamine kinase-a, phosphatidylcholine-specific phospholipase C and D, glycerophosphocholine phosphodiesterase, and several choline transporters in different types of tumor microenvironment is not exactly the same. Changes in these different metabolic pathways have led to differences in choline metabolism (Glunde et al., 2015). Lower concentrations of choline may be an essential nutrient for preventing and protecting degenerative processes such as aging and DNA damage (Merinas-Amo et al., 2017). Choline metabolism also shows an increasingly important position in the diagnosis and treatment of leukemia. The research showed that the choline level of AML patients was lower than that of normal controls, and found that choline may be related to cytogenetic risk (Wang Y et al., 2013). However, in our study, there was no difference in the prognostic grouping (initial diagnosis) based on cytogenetics and gene expression between the AML groups (groups B and C, Table2). 50 patients were statistically analyzed using the prognostic grouping method based on cytogenetics. It was found that there was also no significant statistical difference in the prognosis grouping at the first diagnosis between the AML and ALL groups. This suggests that we have not found that the changes in choline levels are affected by the cytogenetics of leukemia patients in our study, but we are already conducting multi-center cooperation to collect more clinical samples for repeated verification. The phenomenon called “choline phenotype” is related to the occurrence and progression of tumors. It is due to the abnormal choline metabolism caused by the overexpression and over activation of ChoKα, because of the higher levels of phosphorylcholine (PCho) and total bile Alkali compound (tCho) (Glunde et al., 2011). Selective ChoKα inhibitor EB-3D exhibits a potent antiproliferative activity in a panel of T-leukemia cell lines and primary cultures of pediatric patients (Mariotto et al., 2018). This shows that by interfering with choline metabolism, not only can cell apoptosis be induced, but also the sensitivity of T leukemia cells to chemotherapeutic agents (such as dexamethasone and L-asparaginase) can be enhanced (Mariotto et al., 2018). Betaine is a methyl donor formed by oxidation of choline (Roe et al., 2017), it is an isostabilizing amino acid analog. Betaine can cause oxidative stress, cell growth inhibition, inflammation, cell morphological changes and apoptosis in DU-145 cells in a dose-dependent manner (Kar et al., 2019). It can be seen that betaine is also worthy of further exploration of its anti-cancer effect in leukemia. On the one hand, we still collect clinical samples according to strict standards, and will further group them according to strict cytogenetics and gene expression to explore the relationship between them and serum choline and betaine in patients with leukemia, and the stability of choline and betaine as diagnostic markers. We are full of interest in the role and mechanism of betaine and choline in the occurrence and development of leukemia. Considering both the complexity of occurrence and development of tumor, in the subsequent experiments, we will conduct cell experiments to clarify the influence of betaine and choline on the internal and external metabonomics of leukemia cells. At the same time, the leukemia mice model was established, discussed under the condition of the tumor microenvironment, betaine and choline in its role in the development of disease and the corresponding mechanism.

As we all know leukemia is a malignant disease of the blood system, studies have shown that its way of evading tumor immune surveillance is mainly related to the inhibition of natural killer (NK) cells and macrophages (Jaiswal et al., 2009; Al-Matary et al., 2016). ILs have important functions such as promoting lymphocyte differentiation and regulating cell proliferation (Makavos et al., 2020). IL-4 is a pleiotropic cytokine that can regulate a variety of immunological processes under physiological conditions (Kiniwa et al., 2016). Interestingly, IL-4 has two completely opposite effects in regulating mouse phagocytosis, it enhances macrophage-mediated killing of leukemia cells, but also induces CD47 expression, protecting target cells from excessive phagocytosis (Pena-Martinez et al., 2021). IL-10 is a key cytokine that regulates the intensity and duration of the immune response to infection (Couper et al., 2008) studies have shown that its high level is related to the risk of children suffering from ALL. IL-10 gene polymorphism is significantly associated with the susceptibility and pathogenesis of ALL in childhood (Liu et al., 2020). So we used flow cytometry to detect IL-4 and IL-10 in 50 leukemia patients, and compared their differences between different groups. But it was found that there was no significant difference in the levels of IL4 and IL10 between acute lymphoblastic leukemia (group A) and acute myeloid leukemia (group D), so as different groups in AML (group B and C).

Then we compared the differences in laboratory indicators between different leukemia groups. It was found that the lipid profile between the group A (ALL) and D (AML). was different. The triglycerides and high-density lipoproteins of ALL patients were significantly higher than those of AML patients, and the difference was statistically significant, this is consistent with the research of Hina Usman et al. (2015). Hypertriglyceridemia can be used as one of the diagnostic criteria for hemophagocytic lymphohistiocytosis (HLH) associated with certain malignant tumors (Henter et al., 2007). Some studies believe that the degree of lipid abnormalities in patients with ALL and non-Hodgkin’s lymphoma is directly related to the potential tumor burden, especially related to bone marrow involvement (Spiegel et al., 1982). Leukemia patients receiving drug chemotherapy may also affect their blood lipid profile. Studies have shown that the levels of low-density lipoprotein, cholesterol and triglycerides in patients with chronic myeloid leukemia treated with ponatinib for 3 months are significantly increased (Caocci et al., 2020), and the use of l-asparaginase in the treatment of ALL is also associated with severe hyperlipidemia (Bhojwani et al., 2014). A prospective study conducted in children with ALL showed that dyslipidemia returned to normal after completing chemotherapy (Halton et al., 1998). These findings about dyslipidemia suggest that the blood lipid profile of patients with leukemia may be a diagnostic/prognostic factor in the treatment of acute leukemia. In the exploration of leukemia resistance to chemotherapy, some studies have found that renin-angiotensin system (RAS) gene can divide AML patients into different subtypes, and may also be a biomarker of AML drug sensitivity and prognosis (Turk et al., 2020). Excitingly, it has been reported that inhibition of RAS remodels the triacylglycerol network (Sas et al., 2021). This makes our findings worth examining in larger cohorts to determine the association between lipid profiles and leukemia metabolism and the underlying mechanisms. Finally, we further explored the correlation between the differential metabolites of ALL and AML and the patients’ blood lipids and cytokines, and found that there is a significant correlation between the choline and triglycerides. However, there is no significant correlation between the levels of IL-4 and IL-10 and the expression of choline and betaine. Lipid overload may disrupt choline metabolism (Yan et al., 2012). It has been reported that, relative to the effect of placebo, the choline metabolite betaine increases plasma LDL-cholesterol concentration and increases the total cholesterol/HDL-cholesterol ratio (Olthof et al., 2005). In a study on cardiovascular metabolism, it was found that higher plasma phosphatidylcholine concentrations were associated with higher LDL cholesterol and triglycerides (Roe et al., 2017). Because phosphatidylcholine biosynthesis in the liver is involved in the assembly and secretion of lipoprotein particles (Cole et al., 2012). Therefore, our results show that the correlation between triglycerides and choline can be explained. The finding also suggests the mechanism of choline metabolism disorder in leukemia may be closely related to lipid metabolism. We will verify this in subsequent *in vivo* and *in vitro* experiments and conduct in-depth exploration of the mechanism.

In order to better understand the pathways of action of the different metabolites between the above-mentioned different types of leukemia, we carried out KEGG enrichment analysis. As seen in Figures 3G–I, the KEGG enrichment pathways of different metabolites between different types of leukemia are mainly concentrated in amino acid metabolism (glycine and serine metabolism, methionine metabolism), lipid metabolism (phospholipid biosynthesis and beta oxidation of very long chain fatty acids), betaine metabolism and so on. Leukemia cells often rely on specific amino acids for survival, and it is the deficiency of these specific amino acids that provides treatment opportunities for different types of leukemia (Tabe et al., 2019). Amino acids are not only components of proteins but also intermediate metabolites fueling multiple biosynthetic pathways (Tabe et al., 2019). Report shows the proliferation of T-ALL cell lines is dependent on Phosphoserine phosphatase (PSPH), it is generally up-regulated in ALL patients and is associated with high levels of serine and glycine in xenograft mice (Kampen et al., 2019). Serine provides precursors to produce lipids, purines, pyrimidines and antioxidants (Reid et al., 2018). Silence of the phosphoglycerate dehydrogenase (PHGDH) enzyme involved in the serine biosynthesis pathway is detrimental to the growth and survival of leukemia cells (Nguyen et al., 2019). The synthesis of glycine uses serine as an important substrate. It is reported that after restricting the diet of glycine and serine, the growth of colon cancer is inhibited (Maddocks et al., 2013). The study of Barve et al. showed that disturbing the metabolism of methionine and S-adenosylmethionine (SAM) can lead to significant apoptosis and overall changes in cell methylation in AML cells (Barve et al., 2019). Perhaps such a treatment strategy can also be considered in leukemia.

Interestingly, there are reports of a direct correlation between obesity and the recurrence rate of ALL children (Tabe et al., 2019), and fat cells may play an important role in the amino acid metabolism of leukemia. Multiple reports have shown that BM adipocytes treated with asparaginase (ASNase) have an adverse effect on leukemia cells, Gln synthase produced by BM fat cells is up-regulated after chemotherapy, Adipocytes inhibit the cytotoxic activity of ASNase by releasing Glutamine into the leukemia microenvironment (Ehsanipour et al., 2013; Parmentier et al., 2015). ASNase treatment can induce apoptosis by consuming Gln, because cancer cells need a large amount of Gln to maintain TCA replenishment and support cell survival (DeBerardinis et al., 2007). These may further explain the reason for the difference in lipid profile between ALL and AML in this study. We boldly speculate that the difference in lipid profile between ALL and AML patients may be used as an important means of differential diagnosis and treatment entry for lymphocytic leukemia.

Studies have shown that the difference in serum metabolites between leukemia patients and normal controls can be measured because the proliferation of leukemia cells requires lipids, cholesterol and phospholipids (Wojcicki et al., 2020). Our research results are consistent with those of Musharraf, SG and others (Musharraf et al., 2016), choline and lipids are the main substances responsible for the classification of leukemia. Compared with the report of Musharraf, SG et al., we did not only compare the differential metabolites between AML and ALL patients, we also analyzed the correlation between these differential metabolites and common laboratory indicators for monitoring patient condition changes in clinical work. Find the connection between leukemia metabolomics and clinical practice, and establish the direction for its further mechanism research. The abnormal metabolic pathways involved include fatty acid metabolism and lipoprotein changes. In metabolomics, the function of lipids has surpassed membrane composition and energy storage to affect gene regulation and signal transmission (Wojcicki et al., 2020). Experiments have proved that compared with the healthy control group, the expression of steric acid and oleic acid in the acute leukemia group and the acute myeloid leukemia group are up-regulated, while the expression of palmitic acid is down-regulated (Musharraf et al., 2017). The observation results indicate an increase in fatty acid synthesis. It is suggested that the role of fatty acid metabolism is helpful to the diagnosis and treatment of hematological malignancies. After comparing the plasma samples of 8 children with FLT3-ITD and 8 children with wild-type FLT3, it was found that the difference in lysophospholipids was significant. Although the sample size was small, it still suggested that phospholipid metabolism may be related to the imbalance of gene expression (Stockard et al., 2018). Unfortunately, our clinical laboratory indicators do not include genes that affect lipid metabolism, and we will further explore in future studies.

Our findings show that serum metabolomics can successfully differentiate acute lymphoblastic leukaemia from acute myeloid leukaemia, this was also verified with flow cytometry. Admittedly, serum metabolomic analysis does not replace flow cytometry and bone marrow morphology in clinical practice for the identification of leukaemia types. By combining this approach with existing techniques we expect to be able to better differentiate between types of leukaemia. And the metabolism of serum reflects the metabolic changes of the whole body, as the most readily available sample for clinical works; we also hope to gain a better understanding of the physiological characteristics of leukemia. The advantage of our study is that it combines the most common biochemical indicators in clinical work to explore the metabolic characteristics of leukemia, with the expectation that metabolomics will be more easily applicable to clinical work. We present an opportunity to uncover the underlying mechanisms of molecules (choline and betaine) that may differentiate between different leukemia types through lipid profiling. Due to the limited sample size of the single center, we were unable to further enrich the exploration of the metabolic markers that distinguish the respective subtypes in AML and ALL. Leukemia is a very heterogeneous hematological tumor, and there are individual differences in the bone marrow microenvironment. Therefore, even if we have established strict inclusion and exclusion criteria, the impact of tumor heterogeneity on patient metabolism cannot be ruled out. Next, we will further collect more clinical samples to verify the results. We are actively collaborating with other centers and look forward to enriching clinical samples through multi-center collaboration. The study screened out the different metabolites between different leukemias, whether these differential markers can be used clinically as the diagnostic criteria for different types of leukemias needs further confirmation and in subsequent research through targeted metabolomics and the correlation analysis of metabolic differentials with leukemia classification in larger clinical samples. Third, our study only analyzed the differential metabolites between different leukemias. The reason for the differential metabolites between different types of leukemia is still unclear, and the detailed mechanism still needs further study.

## 5 Conclusion

There are obvious metabolic differences between AML and ALL. The metabolism of choline and betaine may also be significantly related to the patient’s blood lipid profile. The main enrichment pathways for distinguishing differential metabolites in different types of leukemia are amino acid metabolism and lipid metabolism, including glycine, serine, arginine, proline, and methionine metabolism, respectively. Differential metabolites and lipid profiles can identify different types of leukemia based on existing clinical diagnostic techniques, and their rich metabolic pathways help us to better understand the physiological characteristics of leukemia.

## Data Availability

The original contributions presented in the study are included in the article/Supplementary Material, further inquiries can be directed to the corresponding author.
