# Supplementary material for: Serum Metabolomics Coupling With Clinical Laboratory Indicators Reveal Taxonomic Features of Leukemia
Source: Front Pharmacol. 2022 May 26;13:794042. doi: 10.3389/fphar.2022.794042 (PMC9204281; doi:10.3389/fphar.2022.794042)
Supplement: Supplementary file 1 [file DataSheet2.PDF]

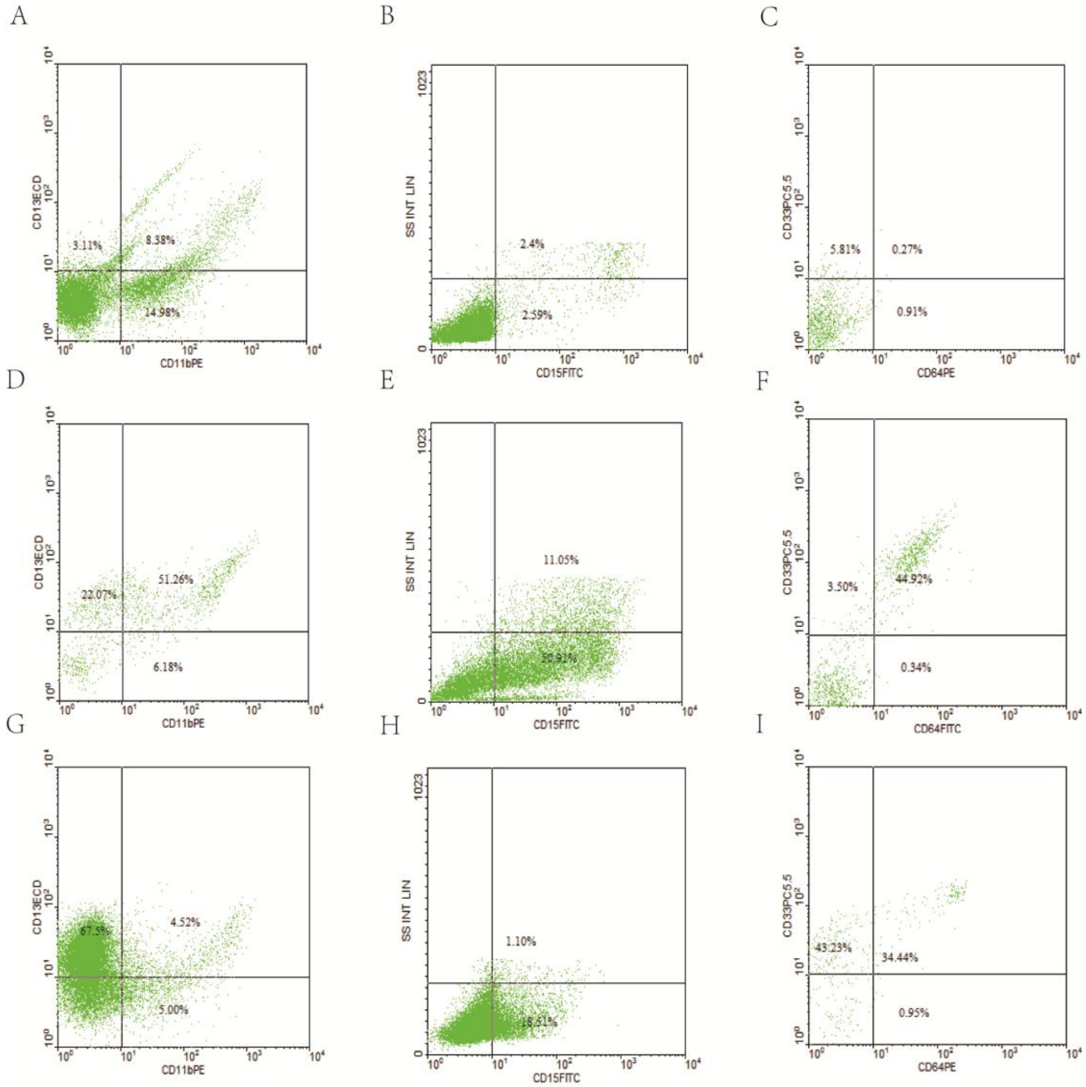

sFigure 1 The expression of leukocyte antigens CD33, CD15, CD64 in each group of cells. A-C acute lymphoblastic leukemia (Group A), D-F acute myeloid leukemia (Group B), G-I acute myeloid leukemia (Group C)
