# Supplementary material for: Serum Metabolomics Coupling With Clinical Laboratory Indicators Reveal Taxonomic Features of Leukemia
Source: Front Pharmacol. 2022 May 26;13:794042. doi: 10.3389/fphar.2022.794042 (PMC9204281; doi:10.3389/fphar.2022.794042)
Supplement: Supplementary file 2 [file DataSheet1.ZIP › Data Sheet 1.PDF]

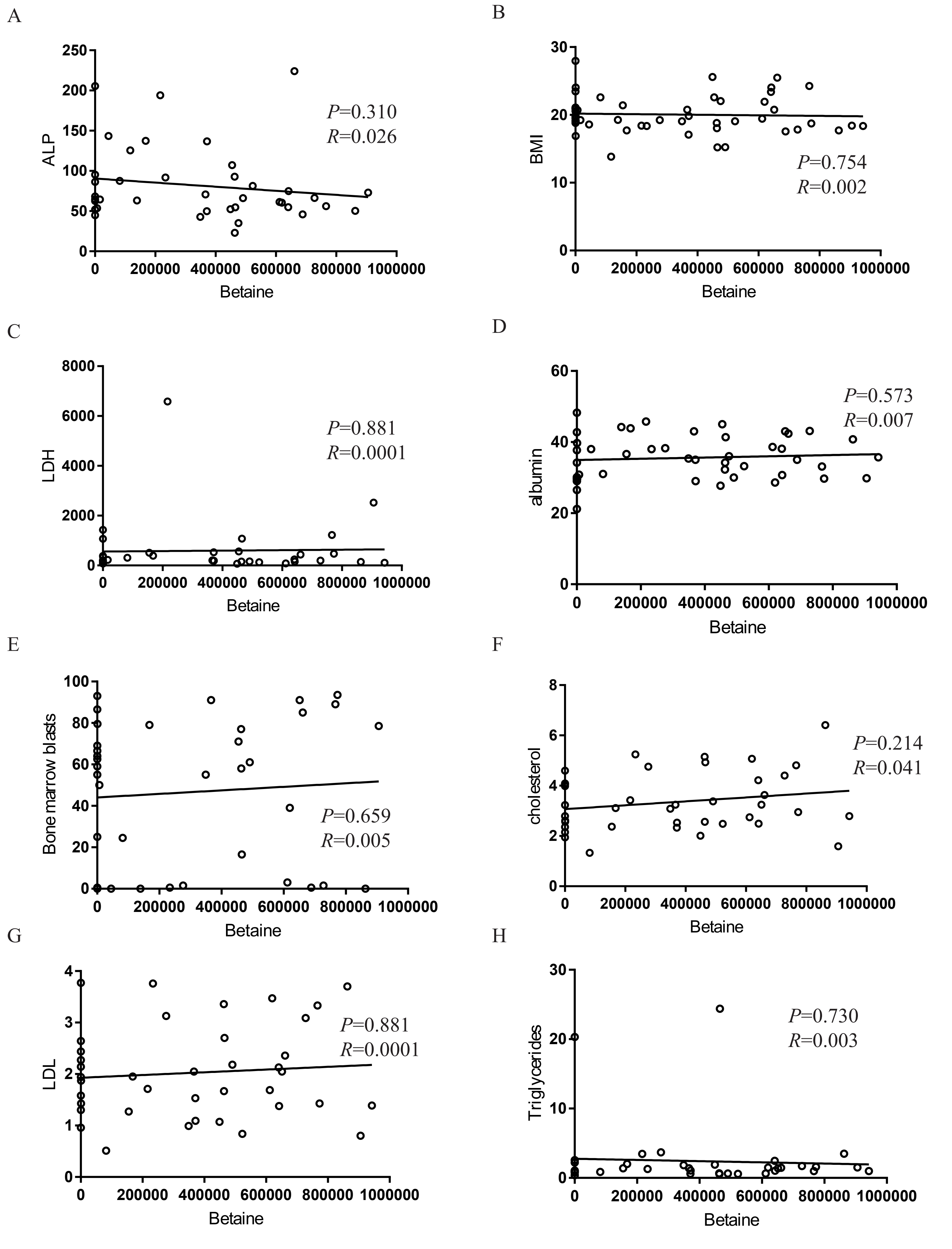

A
ALP
Betaine
P=0.310
R=0.026
B
BMI
Betaine
P=0.754
R=0.002
C
LDH
Betaine
P=0.881
R=0.0001
D
albumin
Betaine
P=0.573
R=0.007
E
Bone marrow blasts
Betaine
P=0.659
R=0.005
F
cholesterol
Betaine
P=0.214
R=0.041
G
LDL
Betaine
P=0.881
R=0.0001
H
Triglycerides
Betaine
P=0.730
R=0.003

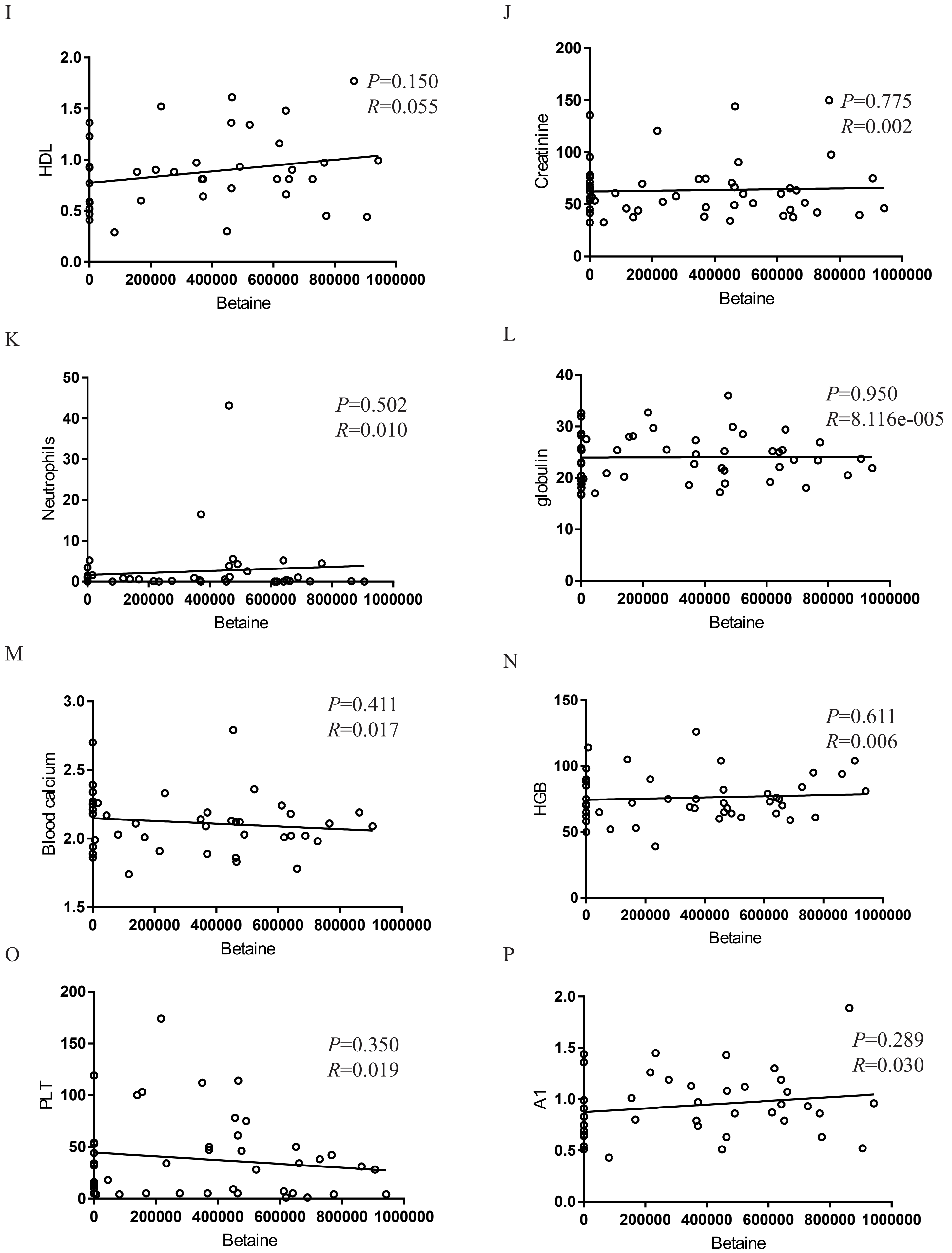
I
HDL
Betaine
P=0.150
R=0.055
J
Creatinine
Betaine
P=0.775
R=0.002
K
Neutrophils
Betaine
P=0.502
R=0.010
L
globulin
Betaine
P=0.950
R=8.116e-005
M
Blood calcium
Betaine
P=0.411
R=0.017
N
HGB
Betaine
P=0.611
R=0.006
O
PLT
Betaine
P=0.350
R=0.019
P
A1
Betaine
P=0.289
R=0.030

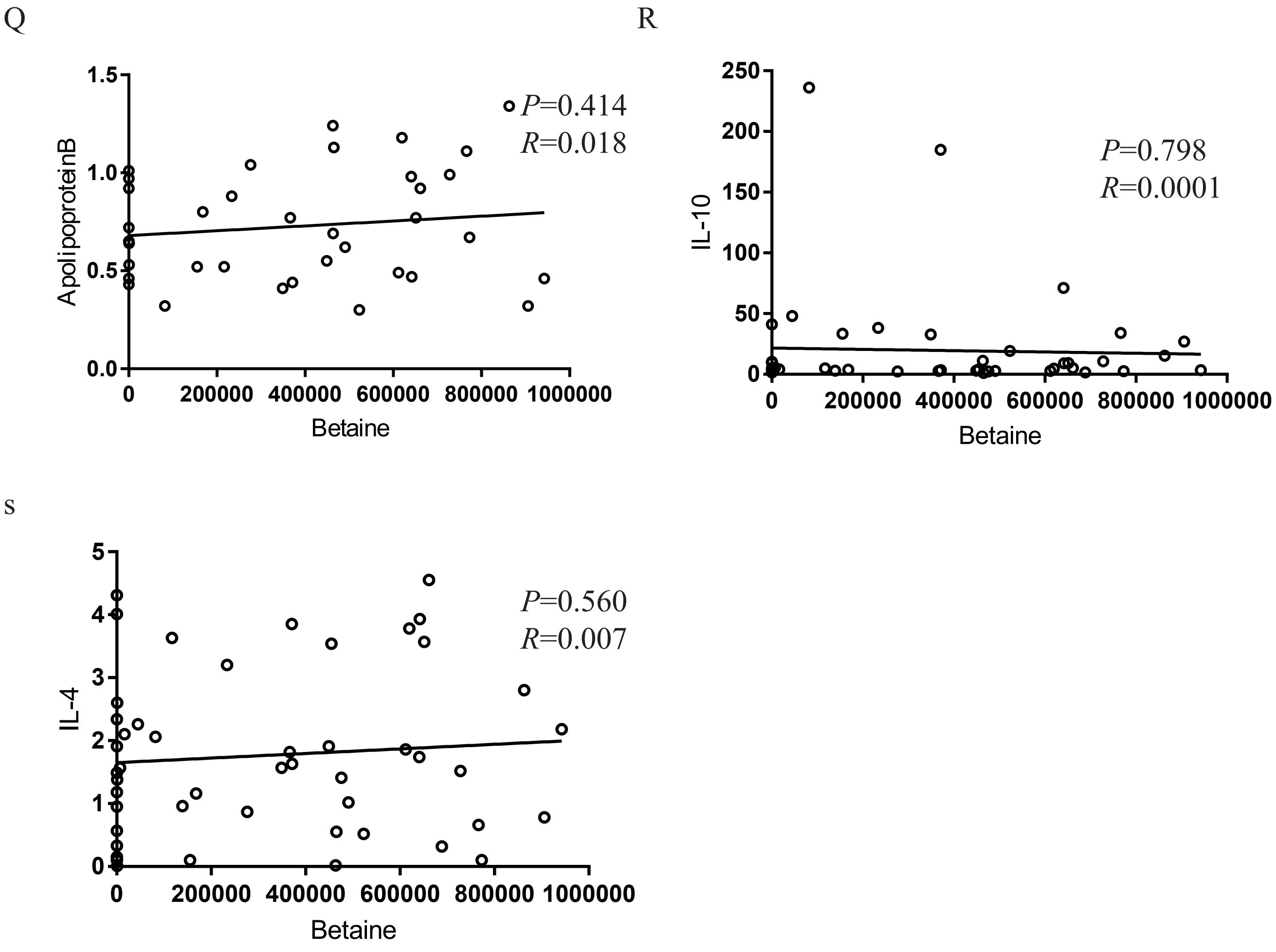

Supplementary Fig. 2 Correlation analysis between Betaine and clinical indicators
