# Supplementary material for: Serum Metabolomics Coupling With Clinical Laboratory Indicators Reveal Taxonomic Features of Leukemia
Source: Front Pharmacol. 2022 May 26;13:794042. doi: 10.3389/fphar.2022.794042 (PMC9204281; doi:10.3389/fphar.2022.794042)
Supplement: Supplementary file 2 [file DataSheet1.ZIP › Data Sheet 2.PDF]

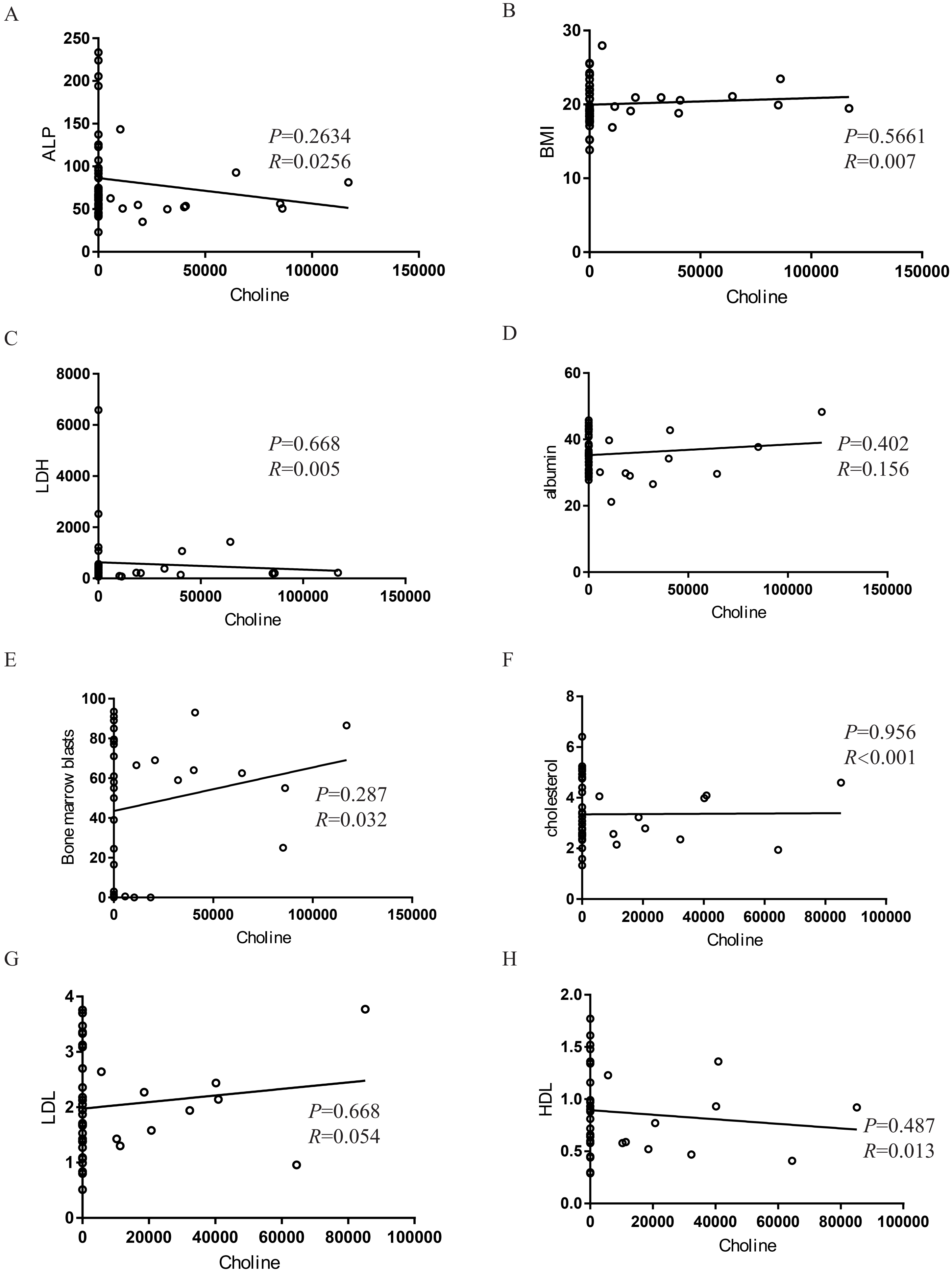
A
ALP
Choline
P=0.2634
R=0.0256
B
BMI
Choline
P=0.5661
R=0.007
C
LDH
Choline
P=0.668
R=0.005
D
albumin
Choline
P=0.402
R=0.156
E
Bone marrow blasts
Choline
P=0.287
R=0.032
F
cholesterol
Choline
P=0.956
R<0.001
G
LDL
Choline
P=0.668
R=0.054
H
HDL
Choline
P=0.487
R=0.013

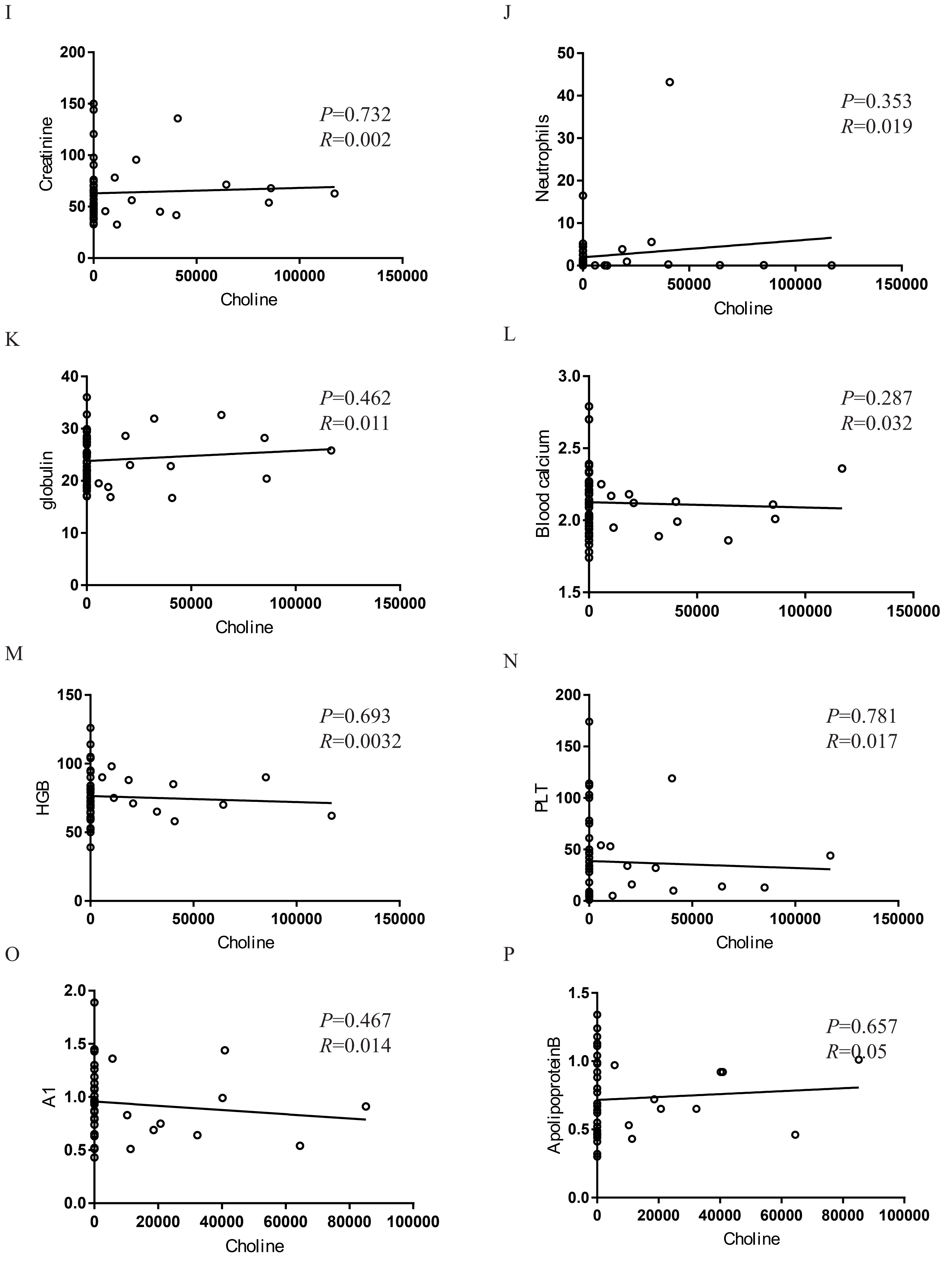

I
Creatinine
Choline
P=0.732
R=0.002
J
Neutrophils
Choline
P=0.353
R=0.019
K
globulin
Choline
P=0.462
R=0.011
L
Blood calcium
Choline
P=0.287
R=0.032
M
HGB
Choline
P=0.693
R=0.0032
N
PLT
Choline
P=0.781
R=0.017
O
A1
Choline
P=0.467
R=0.014
P
ApolipoproteinB
Choline
P=0.657
R=0.05

Q

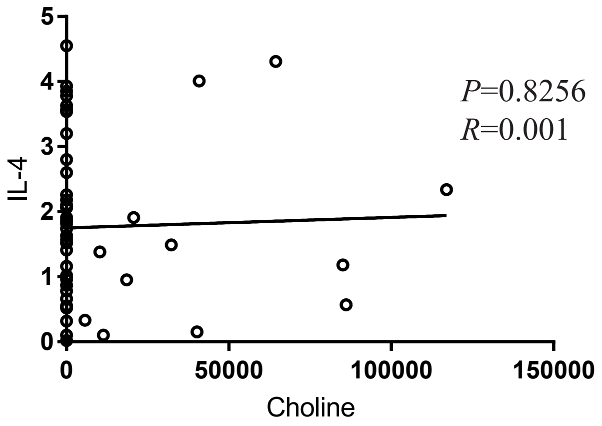

R

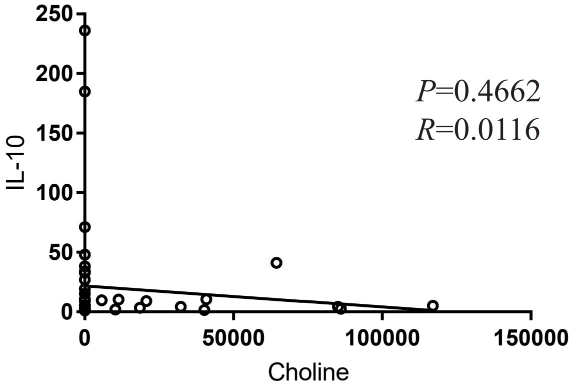

Supplementary Fig. 1 Correlation analysis between choline and clinical indicators
